# Targeting Ergosterol Biosynthesis in *Leishmania donovani*: Essentiality of Sterol 14alpha-demethylase

**DOI:** 10.1371/journal.pntd.0003588

**Published:** 2015-03-13

**Authors:** Laura-Isobel McCall, Amale El Aroussi, Jun Yong Choi, Debora F. Vieira, Geraldine De Muylder, Jonathan B. Johnston, Steven Chen, Danielle Kellar, Jair L. Siqueira-Neto, William R. Roush, Larissa M. Podust, James H. McKerrow

**Affiliations:** 1 Skaggs School of Pharmacy and Pharmaceutical Sciences, University of California San Diego, La Jolla, California, United States of America; 2 Department of Pathology, University of California San Francisco, San Francisco, California, United States of America; 3 Department of Chemistry, Scripps Florida, Jupiter, Florida, United States of America; 4 Department of Pharmaceutical Chemistry, University of California San Francisco, San Francisco, California, United States of America; 5 Small Molecule Discovery Center, University of California San Francisco, San Francisco, California, United States of America; United States Food and Drug Administration, UNITED STATES

## Abstract

*Leishmania* protozoan parasites (Trypanosomatidae family) are the causative agents of cutaneous, mucocutaneous and visceral leishmaniasis worldwide. While these diseases are associated with significant morbidity and mortality, there are few adequate treatments available. Sterol 14alpha-demethylase (CYP51) in the parasite sterol biosynthesis pathway has been the focus of considerable interest as a novel drug target in *Leishmania*. However, its essentiality in *Leishmania donovani* has yet to be determined. Here, we use a dual biological and pharmacological approach to demonstrate that CYP51 is indispensable in *L*. *donovani*. We show via a facilitated knockout approach that chromosomal *CYP51* genes can only be knocked out in the presence of episomal complementation and that this episome cannot be lost from the parasite even under negative selection. In addition, we treated wild-type *L*. *donovani* and CYP51-deficient strains with 4-aminopyridyl-based inhibitors designed specifically for *Trypanosoma cruzi* CYP51. While potency was lower than in *T*. *cruzi*, these inhibitors had increased efficacy in parasites lacking a *CYP51* allele compared to complemented parasites, indicating inhibition of parasite growth via a CYP51-specific mechanism and confirming essentiality of CYP51 in *L*. *donovani*. Overall, these results provide support for further development of CYP51 inhibitors for the treatment of visceral leishmaniasis.

## Author Summary

Visceral leishmaniasis is the second most lethal parasitic infection after malaria. Other forms of leishmaniasis also cause significant morbidity. However, there are few treatments available, and many cause severe side effects or are associated with the development of resistance. A key difference between mammalian cells and *Leishmania* parasites is the type of sterol in their membranes: while mammalian cell membranes contain cholesterol, *Leishmania* parasites use ergosterol. There has therefore been considerable interest in developing inhibitors of sterol biosynthesis pathways to target *Leishmania* parasites. Sterol 14alpha-demethylase (CYP51) is one of the enzymes in the sterol biosynthesis pathway, and the target of significant drug development research in *Leishmania*. Here we use a double approach to determine whether this gene is essential in *Leishmania donovani*, the causative agent of visceral leishmaniasis. We demonstrate via gene knockout and drug targeting approaches that loss or inhibition of CYP51 inhibits *L*. *donovani* growth. These results validate CYP51 as a drug target in *L*. *donovani* and support further work to develop CYP51-directed therapies for visceral leishmaniasis.

## Introduction

*Leishmania* are vector-borne protozoan parasites. They have a digenetic lifecycle; promastigotes are transmitted by the sandfly vector to the mammalian host, where they are taken up by phagocytic cells and differentiate into the amastigote stage within the macrophage phagolysososme. Amastigotes proliferate within the phagolysosome and can be taken up by a sandfly during a subsequent bloodmeal. Within the sandfly gut, amastigotes then differentiate into promastigotes, thereby completing the parasite lifecycle [1].

*Leishmania* parasites cause a range of disease manifestations: cutaneous leishmaniasis in which lesions develop at the site of the sandfly bite, mucocutaneous leishmaniasis with destruction of the mucosal tissues in the nose, mouth and throat, and visceral leishmaniasis in which parasites disseminate to the liver, bone marrow and spleen. Visceral leishmaniasis is the most lethal form of the disease. It is associated with high fever, hepatosplenomegaly and pancytopenia [1]. The infecting species of *Leishmania* is the major determinant of disease manifestation; parasites from the *Leishmania donovani* species complex are the main causes of visceral leishmaniasis, while other species, including the *Leishmania major* species complex, cause cutaneous manifestations [2,3].

*Leishmania* parasites are distributed across tropical and subtropical regions of the world. 350 million people live in endemic areas and are at risk of developing the disease, with 12 million people currently infected [4]. Overall, there are 1.6 million new cases per year [5], associated with a disease burden of 3.3 million DALYs and over 50,000 deaths per year [6], making leishmaniasis the second most lethal parasitic infection after malaria [5]. However, treatment options are limited; while recent progress has been made with the development of single-dose amphotericin B therapy in India [7], this treatment regimen was not effective in East Africa [8]. All other drugs require long treatment regimens; toxicity and drug resistance are also significant concerns [9].

Cell membrane sterols regulate membrane fluidity and contribute to the organization of membrane domains. Unlike mammalian cells, but similar to fungi, *Leishmania* and *Trypanosoma* parasite cell membranes contain ergosterol and ergosterol-like sterols rather than cholesterol. Sterols are generated from acetyl-CoA via a multistep metabolic pathway. The first three steps, catalyzed by acetoacetyl-CoA thiolase, HMG-CoA synthase and HMG-CoA reductase, lead to the generation of mevalonate. Mevalonate is the substrate of the isoprenoid pathway that generates farnesyl diphosphate. Squalene synthase then produces squalene from two farnesyl diphosphate molecules. Squalene is oxidized by squalene oxidase, and the resulting product cyclized to lanosterol. Sterol 14alpha-demethylase (CYP51, LdBPK_111100.1) catalyses the removal of a 14alpha-methyl group from lanosterol [10,11]. The *L*. *infantum* CYP51 enzyme has broad substrate specificity, with the ability to demethylate obtusifoliol, C4-norlanosterol and 14α-methylzymosterol, in addition to lanosterol, although with a preference for the first two substrates [12]. The following steps differ between ergosterol and cholesterol biosynthesis, with variations in the reaction intermediates and enzymes involved depending on species [13]. One of these key latter steps in ergosterol biosynthesis is the methylation of C24 via sterol 24-methyltransferase, leading to the formation of fecosterol, episterol or 5-dehydroepisterol depending on the substrate [14].

Azole antifungals have been investigated for treatment of *Leishmania* infections, but with large variations in efficacy between species [15]. The first experiments on azole sensitivity in visceral *Leishmania* species showed efficacy of ketoconazole [16] and oxiconazole [17] on intracellular amastigotes and of ketoconazole on extracellular promastigotes [18]. Posaconazole [19] and ketoconazole [20] were also effective in mouse models of visceral leishmaniasis, albeit less so than amphotericin B or pentavalent antimonial compounds currently used for visceral leishmaniasis treatments. Azoles have also been extensively tested on cutaneous *Leishmania* species (see for instance [21,22,23,24] for early work on these parasites). Given the importance of CYP51 as a drug target and the severity of disease caused by *L*. *donovani*, we investigated the essentiality of *L*. *donovani* CYP51 by biological and pharmacological methods.

## Materials and Methods

### Ethics statement

All vertebrate animal studies were performed in accordance with the USDA Animal Welfare Act and the Guide for the Care and Use of Laboratory Animals of the National Institutes of Health. The protocol was approved by the University of California San Francisco Institutional Animal Care and Use Committee (protocol AN087316). Euthanasia was performed by carbon dioxide inhalation followed by cervical dislocation.

### Cell culture and infection

*L*. *donovani* 1S/Cl2D promastigotes were maintained at 26°C in M199 medium (Sigma) supplemented with 10% heat inactivated fetal bovine serum (FBS, Sigma), 25 mM HEPES, penicillin, streptomycin, adenosine, glutamine, hemin, and folic acid at pH 7.2. Axenic amastigote differentiation was performed as described in [25]: promastigotes were resuspended in amastigote media (M199 medium supplemented with 25% FBS, streptomycin, penicillin, succinic acid, adenine, glycerol, L-proline and folic acid, at pH 5.5) at a cell density of 5x10^6^ cells/mL and transferred to 37°C, 5% CO_2_.

THP-1 macrophages were maintained in RMPI 1640 media supplemented with 5% FBS 1% penicillin-streptomycin at 37°C, 5% CO_2_. For *Leishmania* infection, THP-1 cells were treated with 50 ng/mL phorbol 12-myristate 13-acetate (PMA) for 48 h and then infected with stationary phase promastigotes. Cells were then fixed with 4% formaldehyde and stained with 4′,6′-diamidino-2-phenylindole (DAPI). Images were obtained with an automated InCell 2000 automated imaging system (G.E. Healthcare) and parasite levels determined using IN CELL developer 1.9 software (see S1 Methods), leading to determination of cell boundaries and counting of parasite inside the boundary but outside the nucleus in an automated fashion.

Female BALB/c mice (17–20 g, 6 per group) were purchased from Simonsen Laboratories and maintained in the animal care facility under pathogen-free conditions. Mice were infected intravenously via the tail vein with 5x10^7^ stationary phase promastigotes and sacrificed 28 days post- infection. Liver parasite burden was determined by direct counting of amastigotes on Diff-Quick stained liver impressions and calculated as Leishman-Donovan Units (LDU): number of amastigotes per 1000 cell nuclei × liver weight (g).

### Generation of transfected *L*. *donovani* lines

All sequences were retrieved from TriTrypDB [26]. 3′ *L*. *donovani CYP51* flanking sequences was amplified by PCR from parasite genomic DNA (LdBPK_111100.1, primers 1 and 2), digested with SpeI and XbaI, and ligated into the XbaI site of vectors pGEM-PAC and pGEM-Hyg. 5′ *CYP51* flanking region was amplified with primers 3 and 4, digested with SpeI XbaI, and ligated into the SpeI site of vectors already containing the *CYP51* 3′ flanking region. Knockout cassettes were then liberated by restriction enzyme digestion with SpeI XbaI. The *CYP51* coding region was amplified by PCR from genomic DNA (primers 5 and 6), digested with BglII and ligated into the BglII site of the pXNG4 vector [27]. In all cases, constructs were verified by diagnostic digest and sequencing.

Transfection was performed as described in [28] by electroporation in cytomix transfection buffer (120mM KCl, 0,15mM CaCl_2_, 10mM K_2_HPO_4_, 25mM HEPES, 2mM EDTA, 2mM MgCl_2_) using a BioRad Gene Pulser Xcell, delivering two pulses at 1500 V and 25 μF. Parasites were transfected first with the hygromycin knockout cassette; HKO clonal lines were selected with 100 μg/ml hygromycin (Invitrogen), then transfected with the empty pXNG4 vector or the pXNG4 vector encoding CYP51, thereby generating the HKO + C and HKO + CYP lines, respectively. Double transfectants were maintained with a combination of hygromycin and 100 μg/ml nourseothricin (GoldBio). HKO + C or HKO + CYP lines were transfected with the puromycin knockout cassette and clonal HKO + C + PAC and HKO + CYP + PAC lines isolated by limiting dilution under selection with hygromycin, nourseothricin and 20 μg/ml puromycin (Sigma). Correct targeting of *CYP51* genes was verified by PCR using primers 7 (in *CYP51* 5′UTR) and 8 (in hygromycin resistance gene) or 9 (in puromycin resistance gene). Persistence of *CYP51* genes in double resistant, uncomplemented strains and loss of chromosomal *CYP51* in complemented strains were verified by PCR (primers 10 + 11 and primers 12 + 13, respectively). Primer 12 is upstream of *CYP51*, outside of the knockout cassette, and primer 13 anneals within the *CYP51* gene.

### Ganciclovir selection

50 μg/mL ganciclovir (Invivogen) was added to the parasite cultures. qPCR on extracted DNA to monitor pXNG4 loss and flow cytometry analysis to assess GFP levels were performed weekly (see below). Results shown represent the average of two independent selection experiments on a total of seven independent clonal lines.

### Quantitative PCR

DNA was extracted as described previously [29]. qPCR reactions containing 100 ng of parasite DNA in Lightcycler 480 Sybr green I Master mix (Roche) were run on a Stratagene Mx3005P RT-PCR thermocycler using the following thermal profile: initial denaturation at 95°C for 10 min, then 40 cycles of denaturation at 95°C for 10 s, annealing at 57°C for 20 s and extension at 72°C for 20 s. Melting curve analysis and agarose gel electrophoresis were used to confirm correct PCR product formation. Chromosomal *CYP51* (primers 14 and 15), total *CYP51* (primers 16 and 17), and pXNG4 (primers 18 and 19) relative levels were determined by qPCR using the 2^-ΔΔCt^ method [30], normalizing to serine acetyltransferase (SAT, primers 20 and 21) or cystathionine beta-synthase (CBS, primers 22 and 23), previously shown to be present in only two copies in *L*. *donovani* [31].

### Flow cytometry

Analyses were performed on a BDFACSDiva LSRII flow cytometer in HTS mode. Cells were stained with 5 μM propidium iodide (PI, Sigma). Quadrant gates were set used PI-stained wild-type parasites (GFP-negative) and percentage of GFP+ PI- cells determined using FloJo X software (Tree Star Inc).

### SDS PAGE and Western blot

1x10^7^ parasites were lysed in 1x LDS buffer (Invitrogen) and separated using NuPage bis-tris precast polyacrylamide gels (Invitrogen). Proteins were transferred to a PVDF membrane (BioRad). Western blot was performed as described in [32]. Affinity-purified anti-CYP51 antibodies (Genescript) and anti-GAPDH antibody (from Paul Michels, Université catholique de Louvain, Bruxelles) were used at 1:5,000 dilution. The secondary antibody was a 1:5,000 dilution of peroxidase-conjugated anti-rabbit IgG antibody (GE Healthcare). All proteins were visualized using SuperSignal West Pico Chemoluminescent Substrate (Thermo Scientific). Proteins expression levels were quantified with Image J program, normalizing CYP51 levels to GAPDH levels.

### Sterol GC-MS

Sterol extraction was performed as described in [33]. Briefly, the parasite cell pellet was resuspended in chloroform-methanol solution (2:1 ratio), then dried under nitrogen gas, followed by overnight treatment with chloroform. The organic phase was then washed with water and dried under nitrogen. The dried pellet was resuspended in chloroform-methanol (9:1 ratio), and washed again with water. Acetonitrile was added to the samples, washing steps were repeated and solvents evaporated under nitrogen.

Extracted sterols were then derivatized by resuspending the dried residue in 25 μL hexanes and 75 μL BSTFA (Sigma-Aldrich, St. Louis MO) for 2 hr at 37°C to generate the trimethylsilyl (TMS) sterols. TMS-derivatized sterols were analyzed using gas chromatography-mass spectrometry (GC-MS) on an Agilent HP 6850 GC coupled to a mass selective detector (Agilent MSD 5973) operating at 70 eV in electron impact mode. The sterols were separated using a DB5-MS analytical column (30 m x 0.25 mm inner diameter, 0.25-μm film thickness, Agilent) with a temperature profile that begins at 200°C for 1 min, increases by 15°C/min up to 300°C, and holds at 300°C for 20 min. The inlet and detector temperatures were held at 200 and 250°C, respectively. The MSD was set to scan the range 50–750 *m/z* for sterol profiling. Selected Ion Monitoring (SIM) was used for ergosterol quantification by using the same GC temperature profile but assaying for fragment ions specific to ergosterol that elute at the same time window as ergosterol standard: *m/z* 468.4, 378.4, 363.4, 337.4, and 253.1 We prepared an 8-point standard curve of ergosterol using serial dilution over a concentration range of 9 pmol to 1.2 nmoles. The area under the curve in the SIM assay was then compared to standard samples to calculate ergosterol concentrations.

### Ergosterol biosynthesis inhibitor assay

Amphotericin B, ketoconazole and voriconazole were purchased from Sigma. All other CYP51 inhibitors were synthesized in-house (see supplementary methods and [34,35,36]). Stationary phase promastigotes (8x10^5^/mL) were treated for 72 h with two-fold dilution of inhibitors in 384 well plate format. Resazurin (0.025 mg/mL, Santa Cruz) was added for 5 h, cells were fixed, and fluorescence measured at 490 nm excitation and 595 nm emission wavelengths. Data was normalized to the amphotericin B positive control and DMSO vehicle negative control for each plate, and EC50 values calculated using Collaborative Drug Discovery Vault software. *T*. *cruzi* cell-based activity was determined by high content screening in triplicate, as previously described [36].

## Results

### *Leishmania donovani* tolerate modulations in CYP51 levels

**Fig 1:**
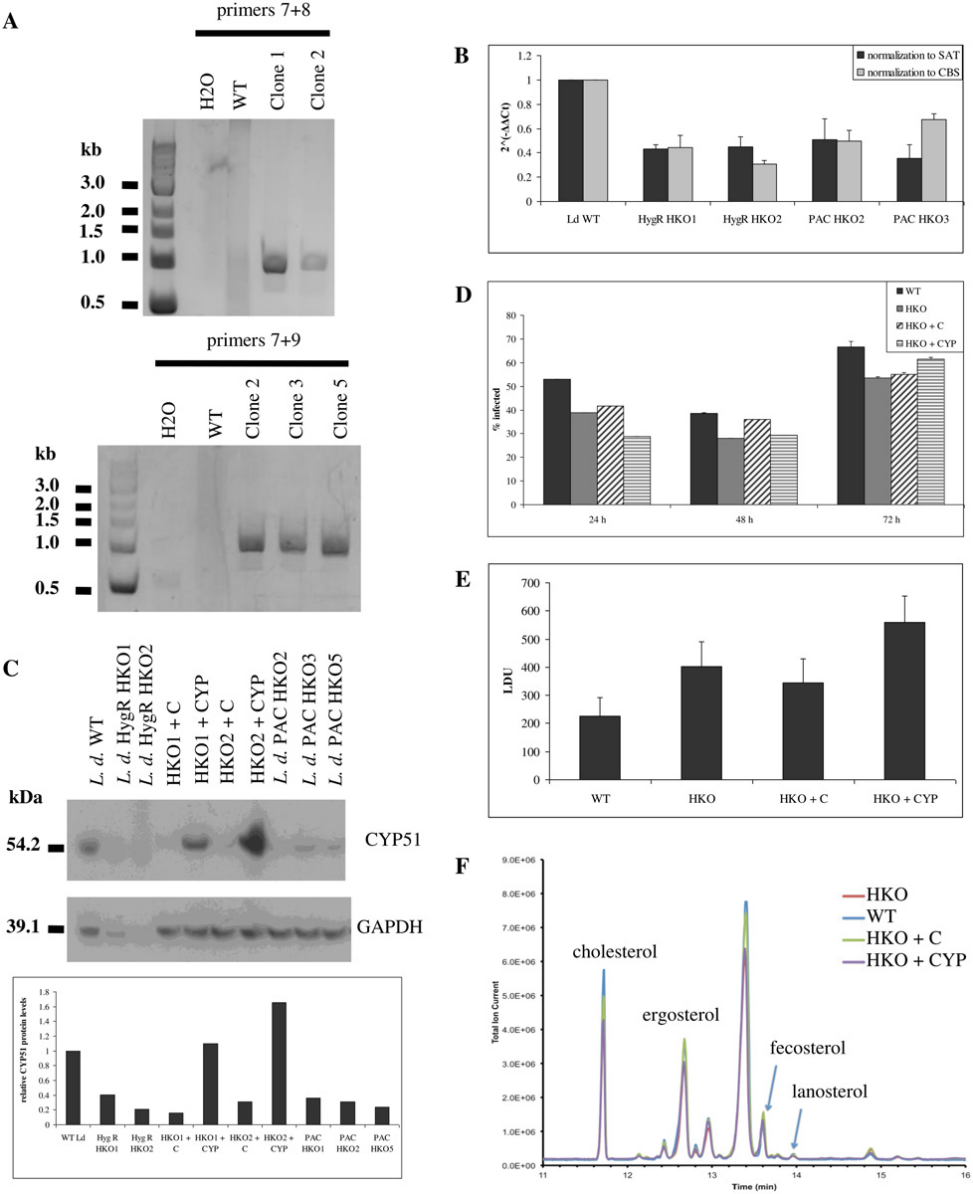
Modulation of CYP51 levels in *L*. *donovani*. **A**, Replacement of a *CYP51* allele by homologous recombination. Correct targeting of the knockout cassettes was verified by PCR using one primer within the knockout cassette and one upstream of *CYP51* (primers 7 and 8 (hygromycin), top or 7 and 9 (puromycin), bottom). **B**, qPCR quantification of chromosomal *CYP51* levels, normalized to SAT or to CBS and to wild-type levels. **C**, CYP51 protein levels in half knockout and complemented strains. CYP51 and GAPDH were detected by Western blot (top) and quantified by densitometry (bottom) **D**, *In vitro* infectivity of half knockout and complemented strains. THP1 macrophages were infected at a 10:1 parasite to macrophage ratio. Cells were fixed and stained with DAPI 24, 48 and 72 h post-infection, and macrophage infection levels were determined by automated high-throughput imaging and parasite detection. **E**, *In vivo* infectivity of half knockout and complemented strains. BALB/c mice were infected intravenously. Liver parasite burden (Leishman-Donovan Units, LDU) was determined 28 days post-infection by counting stained liver impressions. **F**, Sterol profiling by GC-MS.

**Fig 2:**
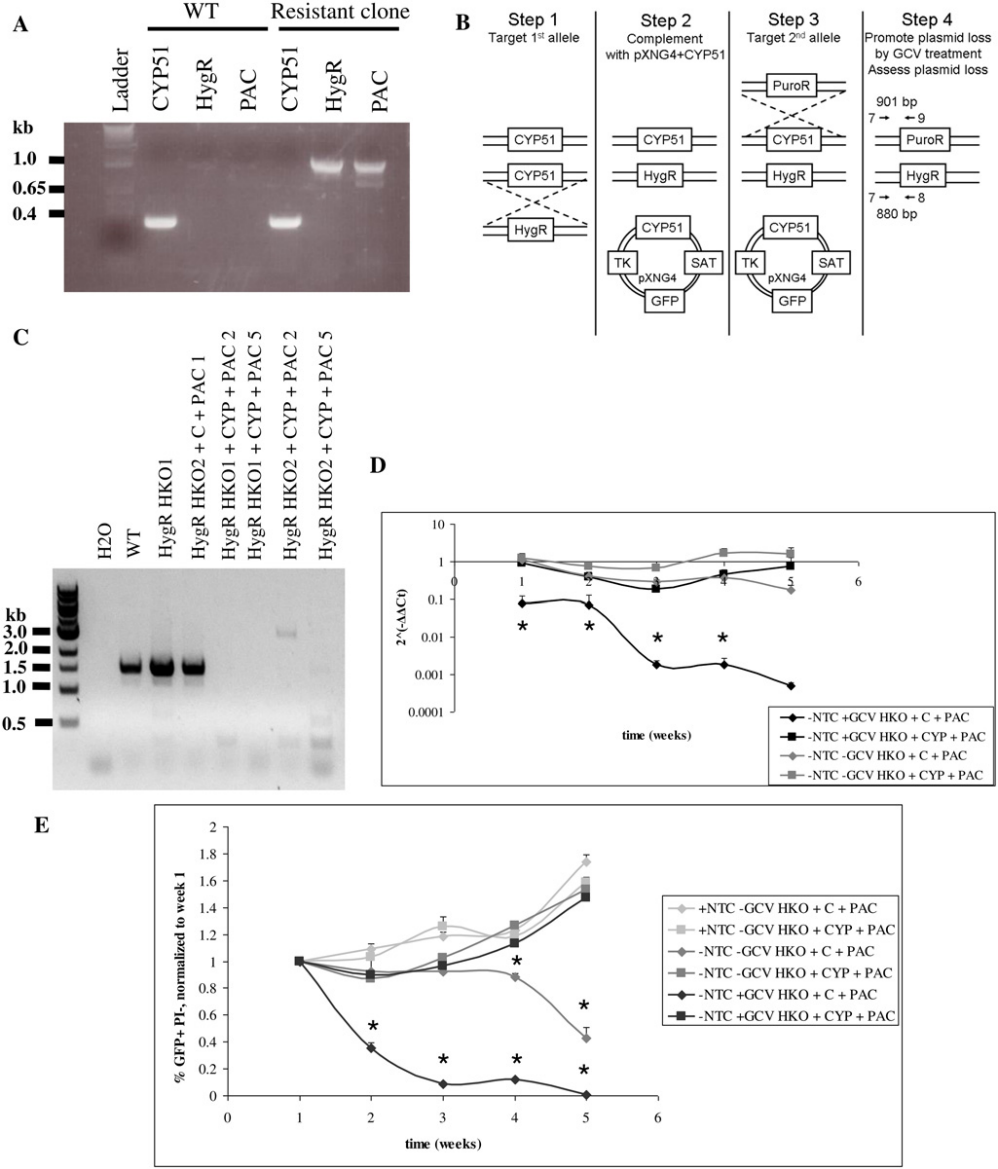
Essentiality of CYP51 in *L*. *donovani*. **A**, Persistence of *CYP51* gene even after correct targeting of two knockout cassettes into the *CYP51* locus. Correct targeting of the knockout cassettes was verified by PCR using one primer within the knockout cassette and one upstream of *CYP51* (primers 7 and 8 (hygromycin) or 7 and 9 (puromycin)). *CYP51* was amplified with primers within the coding region (primers 10 and 11). **B**, Targeting strategy. Primers 7 and 8 or 7 and 9 and expected PCR product sizes to confirm correct targeting of chromosomal CYP51 alleles are indicated by arrows (step 4). **C**, Loss of chromosomal *CYP51* in the presence of episomal *CYP51* complementation but not in strains complemented with the empty vector. PCR was performed using primers 12+13. **D**, pXNG4 loss monitored by qPCR during GCV selection or in the absence of either positive or negative selection (untreated samples). pXNG4 Ct values were normalized to SAT levels and to samples under positive selection (treated with nourseothricn (NTC) in the absence of GCV). **E**, GFP positive, propidium iodide (PI) negative levels were monitored by flow cytometry. Percentage of GFP+ PI- cells were normalized to the levels for week 1. *, p<0.05 compared to HKO+CYP+PAC.

We generated half knockout *L*. *donovani* parasites (HKO strains) in which a single *CYP51* allele was replaced with either a puromycin or hygromycin resistance marker (Fig. 1A; see Fig. 2B for the knockout approach). *CYP51* is located on chromosome 11, which is disomic in reference *L*. *donovani* genomes [37], but trisomic in some clinical *L*. *donovani* isolates [31]. In addition, the *Leishmania* genome contains many direct and indirect repeats that can promote extrachromosomal element formation under drug pressure or for essential genes [31,38]. Prior to targeting another *CYP51* allele, we therefore verified *CYP51* copy number in parasites transfected with the first knockout cassette, resistant to either hygromycin (HygR HKO strains) or puromycin (PAC HKO strains). HKO strains contained half of the *CYP51* DNA content found in wild-type, indicating loss of one out of two alleles (Fig. 1B). Furthermore, CYP51 protein levels were decreased two to five fold in half knockout strains. Complementation with an episomal *CYP51* gene restored protein expression to levels comparable to wild-type *L*. *donovani* (Fig. 1C).

Given the importance of ergosterol biosynthesis in trypanosomatid parasites, we assessed the impact of this loss of CYP51 expression before proceeding to targeting of the second *CYP51* allele. *In vivo* and *in vitro* infectivity was comparable between strains (Fig. 1D, Fig. 1E, S1 Fig); any differences between wild-type and transfected strains were not due to changes in CYP51 levels since infectivity of HKO, HKO+C and HKO+CYP was comparable. These strains also all had comparable sterol profiles (Fig. 1F) and ergosterol levels (Table 1).

**Table 1:** Ergosterol levels in half knockout strains.

Stage	Strain	Ergosterol levels (pmol)^a^
**Promastigotes**	Wild-type	7236.7
	HKO	5167.3
	HKO + C	7535.2
	HKO + CYP	5887.9
**Axenic amastigotes**	Wild-type	1374.7
	HKO	1102.8
	HKO + C	904.4
	HKO + CYP	983.3
**Intracellular amastigotes**	Wild-type	135.2
	HKO	165.3
	HKO + C	462.0
	HKO + CYP	327.7

^a^ Normalized to host cholesterol levels for intracellular amastigotes

### Essentiality of CYP51: Biological approach

Since *L*. *donovani* parasites were able to tolerate over two-fold reductions in CYP51 protein levels with no apparent effects on parasite phenotype, we then proceeded to targeting the second *CYP51* allele. However, while we obtained correct targeting of *CYP51* with hygromycin and puromycin resistance markers, double drug-resistant parasites still retained *CYP51*, despite multiple targeting attempts (Fig. 2A). We therefore used a facilitated knockout approach (Fig. 2B) [27,39,40], targeting the second *CYP51* allele in the presence of episomal *CYP51* complementation. As a control, we targeted this second allele in parasites transfected with the empty pXNG4 vector [27] (S2 Fig.). We obtained complete loss of chromosomal *CYP51* genes only in the presence of episomal *CYP51*; genomic *CYP51* was retained in the parasites transfected with the empty plasmid, supporting essentiality of *CYP51* (Fig. 2C).

The pXNG4 vector used for complementation also encodes a green fluorescence protein gene (GFP) and a herpes virus thymidine kinase gene; cells that contain the plasmid are sensitive to treatment with ganciclovir (GCV). We therefore performed negative selection against the pXNG4 vector by treating transfected parasites with GCV. Plasmid persistence during GCV treatment was monitored by qPCR and by flow cytometry for GFP. The pXNG4 plasmid was lost much faster from parasites transfected with the empty vector (retain chromosomal *CYP51*) than from parasites transfected with the vector encoding CYP51 (only source of *CYP51*), indicating selection for *CYP51* persistence (Fig. 2D, 2E). One clonal line (HKO1 + CYP + PAC2) showed greater loss of pXNG4 plasmid, but still retained *CYP51* DNA levels comparable to half knockout strains, with 2^(-ΔΔCt) values of 0.6, leading to ergosterol levels similar to wild-type, even after 7 weeks of GCV selection (S4 Fig). Overall, these results support essentiality of CYP51 in *L*. *donovani*.

### Essentiality of CYP51: Pharmacological approach

**Fig 3:**
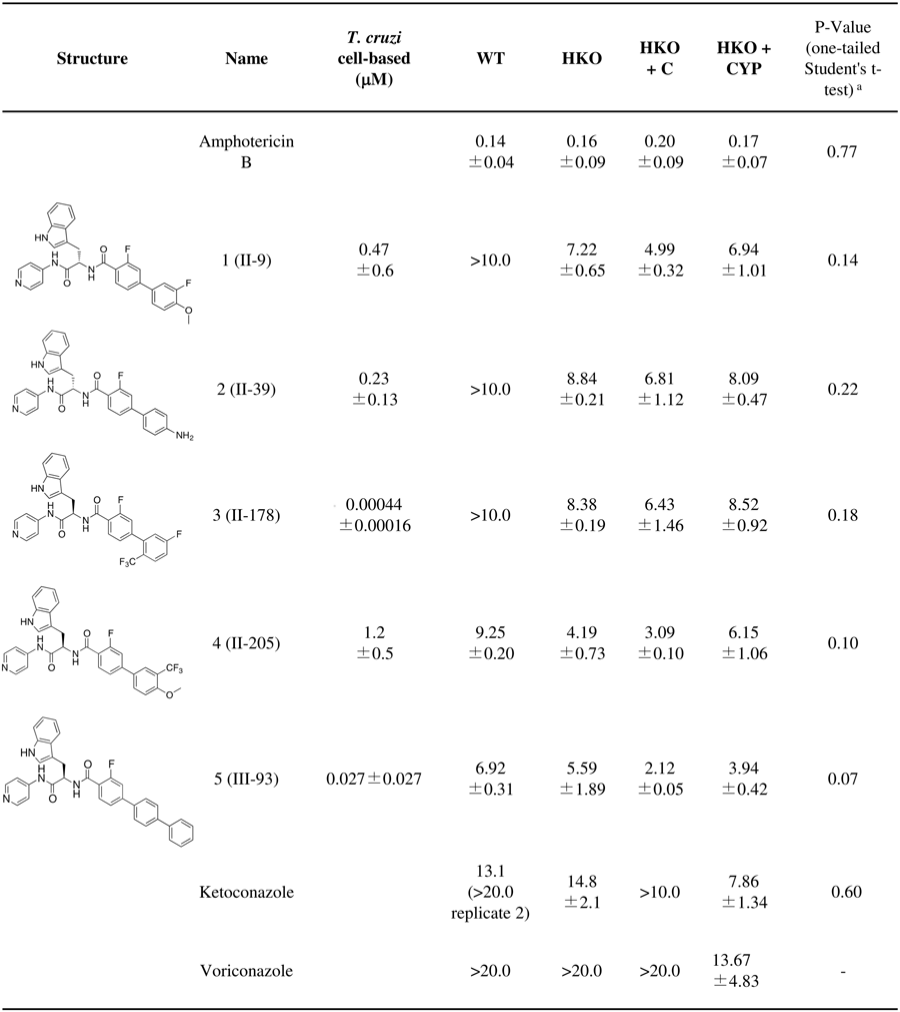
Susceptibility of half knockout strains to CYP51 inhibitors (EC50, μM): compounds with no significant differences between HKO + C and HKO + CYP strains. Values ± standard error are shown. ^a^ p-values are for comparison between HKO+C and HKO+CYP.

**Fig 4:**
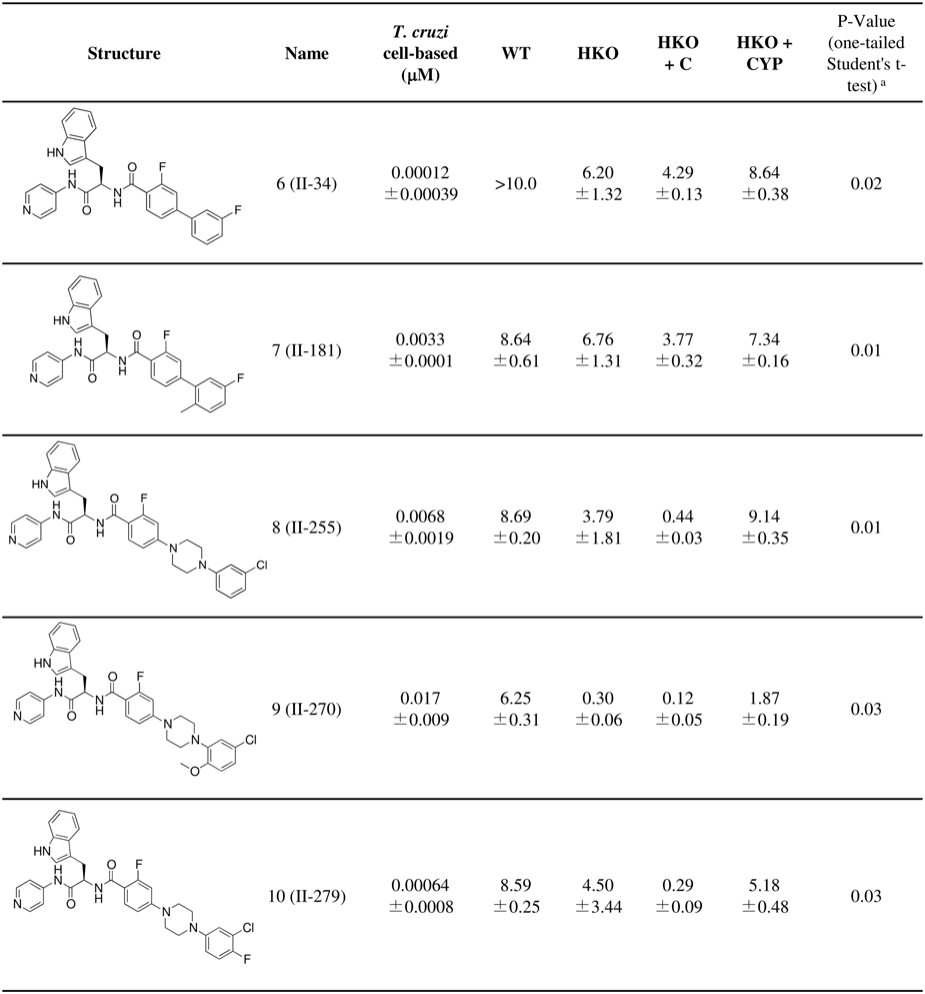
Susceptibility of half knockout strains to CYP51 inhibitors (EC50, μM): compounds with decreased activity on HKO + CYP strains. Values ± standard error are shown. ^a^ p-values are for comparison between HKO+C and HKO+CYP.

The persistence of CYP51-encoding pXNG4 plasmids even under GCV negative selection indicates that CYP51 is essential in *L*. *donovani*. Pharmacological inhibition of CYP51 should therefore lead to parasite growth arrest and death. The 4-aminopyridyl-based compound series of CYP51 inhibitors was derived from an initial hit in target-based high-throughput screening, followed by hit-to-lead optimization using structure-activity relationships (SAR), structure-property relationships (SPR), and biological and structural evaluation for *T*. *cruzi* CYP51 [34,35,36,41,42,43,44]. We tested 205 compounds from this series on wild-type intracellular *L*. *donovani* amastigotes by high content assay. Fifty-four compounds with over 60% activity at 10 μM were then used for dose-response experiments on wild-type *L*. *donovani* promastigotes and strains in which we modulated CYP51 expression (HKO, HKO+C and HKO+CYP). Representative compounds with the highest activity on promastigotes are shown in Fig. 3, Fig. 4. Activity on intracellular amastigotes is shown in S3 Table. No clear difference in EC50 values were observed between strains with ketoconazole and voriconazole controls, possibly due to their lower activity on *L*. *donovani*. In-house compounds were more potent in this assay than the commercial antifungal azoles. Overall, HKO+CYP strains were less sensitive to these 4-aminopyridyl-based inhibitors compared to HKO+C, indicating that these compounds inhibit *Leishmania* growth via a CYP51-mediated mechanism. This confirms that targeting CYP51 pharmacologically promotes inhibition of parasite growth, further supporting essentiality of CYP51 in *L*. *donovani* metabolism.

## Discussion

CYP51 (sterol 14alpha-demethylase) belongs to the large cytochrome P450 enzyme family, which contains over 20,000 members. While there is significant variation at the sequence level, CYP51 is highly conserved across eukaroytes at the structural level [45]. However, small variations between species and strains can lead to significant variations in sensitivity to CYP51 inhibitors. Indeed, a single amino acid change in CYP51 in *T*. *cruzi* Y strain compared to Tulahuen strain was associated with significant decrease in sensitivity to two CYP51 inhibitors, at concentrations that caused 100% inhibition of the Tulahuen enzyme [46].

*In vitro* side-by-side comparison of azole efficacy on promastigotes between *Leishmania* species provides conflicting results: two studies observed increased susceptibility of six different *L*. *donovani* strains to ketoconazole and itraconazole compared to six different *L*. *major* strains [14,18], while other studies on different *L*. *donovani* and *L*. *major* strains indicated that *L*. *donovani* is more resistant to ketoconazole [47] and posaconazole [19] than *L*. *major*. In a separate study, intracellular *L*. *donovani* amastigotes were more sensitive to ketoconazole than amastigotes from cutaneous leishmaniasis patients [16]. *L*. *major* promastigotes were also insensitive to our 4-aminopyridyl-based compound series of CYP51 inhibitors, even with a longer exposure to the compounds (S2 Table). With regards to clinical trials, azoles have shown large variations in clinical efficacy between *Leishmania* species, from no effect to almost 90% efficacy [15], although the majority of these studies have focused on cutaneous leishmaniasis. While there is a single case report of successful posaconazole use to treat cutaneous leishmaniasis caused by *L*. *donovani infantum* [48], to our knowledge there has been no clinical trial of azoles for visceral leishmaniasis. Persistent *Leishmania* growth in the presence of azoles has been tied to tolerance to 14-methyl sterol accumulation in parasite membranes [14,18,49] as well as increased exogenous cholesterol incorporation [18].

Recent work indicated that CYP51 appears to be dispensable in *L*. *major*, albeit at a high fitness cost [14]. In contrast, given (1) our inability to fully knockout chromosomal *CYP51* unless we provide an extrachromosomal episomal source of *CYP51*, (2) the persistence of this *CYP51* episome during negative selection under conditions in which it is the only source of *CYP51*, and (3) the CYP51-specific growth inhibition of 4-aminopyridyl-based non-azole CYP51 inhibitors in *L*. *donovani*, our results support essentiality of CYP51 in *L*. *donovani*. While extrachromosomal episomal-encoded CYP51 could not fully complement the knockout phenotype, it was indeed active, given its ability to substitute for chromosomal CYP51 and to increase resistance to the 4-aminopyridyl-based non-azole CYP51 inhibitors which directly target the CYP51 active site [13,34,36].

*L*. *donovani* and *L*. *major* CYP51 are overall very similar. Comparing the *L*. *major* and *L*. *donovani* CYP51 protein sequences highlights two amino acid substitutions in β helices 1–1 and 1–2 and a single amino acid insertion at the C-terminal in *L*. *donovani* compared to *L*. *major* (S5 Fig). This suggests that other mechanisms may be responsible for the observed differences in CYP51 essentiality between *L*. *major* and *L*. *donovani*. Indeed, squalene synthase, which catalyzes the first committed step in ergosterol biosynthesis, has been involved in resistance to itraconazole [50]; differences in sensitivity to some squalene synthase inhibitors were observed between *L*. *major* and *L*. *donovani* [51]. Likewise, there were differences in sensitivity to sterol 24-methyltransferase inhibitors between *L*. *major* and *L*. *donovani* [52]. Finally, the activity of *L*. *donovani* 3-hydroxy-3-methylglutaryl coenzyme A reductase (HMG-CoA reductase) was 50-fold higher than the activity of the *L*. *major* enzyme [53]. HMG-CoA reductase catalyzes the third step of sterol synthesis from acetyl-CoA and is the rate-limiting step in human sterol biosynthesis [54]. Finally, another member of the cytochrome P450 family, CYP5122A1 (LdBPK_270090.1), also modulates ergosterol levels in *L*. *donovani* [55]; its expression or activity could be altered in CYP51-deficient *L*. *major*, to complement for loss of CYP51.

Beyond differences in CYP51 and sterol biosynthetic pathways between *L*. *major* and *L*. *donovani*, additional factors could also contribute to this observed difference in CYP51 essentiality. Indeed, Xu *et al* showed that *L*. *major* CYP51 is involved in protection against heat shock [14]. *L*. *major* is considerably more sensitive to heat shock than *L*. *donovani*, but the mechanism of resistance to heat shock differs between these species, with the *L*. *donovani-*specific A2 protein family a key contributor to *L*. *donovani* survival during heat stress [25,32]. Likewise, gp63 and lipophosphoglycan levels were altered in CYP51-deficient *L*. *major* [14]. Lipophosphoglycan is structurally different between *L*. *major* and *L*. *donovani* [56], and gp63 from members of the *L*. *donovani* species complex is less active than *L*. *major* gp63 [57].

Overall, our results support further investigation of CYP51 inhibitors for the treatment of visceral leishmaniasis. While recent clinical trial results using posaconazole for the treatment of Chagas disease were disappointing [58], the enhanced potency we observed in *L*. *donovani* for 4-aminopyridyl-based non-azole inhibitors of CYP51 compared to ketoconazole and voriconazole supports the development of novel inhibitor scaffolds, potentially using our 4-aminopyridyl inhibitor series as a starting point. Given the lower efficacy of these inhibitors on *Leishmania* compared to *T*. *cruzi*, efforts should be made through further medicinal chemistry to optimize both pharmacodynamic and pharmacokimetic properties of these compounds for activity against *Leishmania*. In particular, efficacy was much lower for intracellular wild-type *L*. *donovani* amastigotes compared to our transfected promastigote strains (S3 Table), possibly due to additional constraints with regards to drug uptake into the host cell and into the parasite-containing acidic phagolysosome. Finally, this work and the work of others indicate that CYP51-targeted therapies may not be suitable to treat all *Leishmania* species. This highlights the importance of considering variations between species and strains early during the drug development process.

## Supporting Information

S1 Methods Supplemental methods. (DOC)

S1 Scheme Synthesis of CYP51 inhibitors. Reagents and conditions: (a) Arylboronic acid, 5 mol% Pd_2_(dba)_3_, 10 mol% PCy_3_, 2M K_3_PO_4_, dioxane, 100°C (microwave), 1h, ca. 90% (b) 1-(aryl)piperazine, Pd(OAc)_2_, P(o-tolyl)_3_, Cs_2_CO_3_, toluene, 50°C, 48 h, ca. 70% (c) 10% NaOH (aq), MeOH/THF (1/1), 60°C, 3 h, ca. 95% (d) **11, 12, 13**, **14**, **15**, **16**, **17**, **18b**, **19b**, and **20b** (as appropriate), PyBOP, HOBt, Et_3_N, CH_2_Cl_2_, 23°C, 1h, ca. 50%.
(PPT)

S1 Fig *In vitro* infectivity of half knockout and complemented strains. THP1 macrophages were infected at a 10:1 parasite to macrophage ratio. Cells were fixed and stained with DAPI 24, 48 and 72 h post-infection, and parasite numbers per infected cell were determined by automated high-throughput imaging and parasite detection
(PPT)

S2 Fig Targeting of hygromycin and puromycin resistance knockout cassettes. Correct targeting of the hygromycin (**A**) and puromycin (**B**) resistance knockout cassettes was verified by PCR using one primer upstream of *CYP51* and one specific to the resistance marker (primers 7 and 8 (hygromycin) or 7 and 9 (puromycin)). HKO1 + CYP + PAC clones 2, 4, 5; HKO2 + C + PAC clones 1, 2 and 3; HKO2 + CYP + PAC clones 1, 2, 4 and 5 have correct targeting of both knockout cassettes.
(PPT)

S3 Fig Representative flow cytometry analysis at five weeks of GCV selection. Parasites were treated with NTC (positive selection), GCV (negative selection) or left untreated (-NTC-GCV) for five weeks. One representative cell line is shown for HKO + C + PAC and for HKO + CYP + PAC. **A**, Quadrant analysis. Numbers indicate the percentage of cells in each quadrant. **B**, Representative GFP histogram plots of PI-negative cells. Wild-type parasites (dotted line) serve as the non-fluorescent cutoff reference. Black, NTC treatment (positive selection). Grey, GCV treatment (negative selection).
(PPT)

S4 Fig Persistence of CYP51 in HKO1 + CYP + PAC2 strain. CYP51 persistence was assessed by qPCR (**A**) and Western blot (**B**) following seven weeks of GCV selection. Sterol profiles and ergosterol levels were determined by GC-MS (**C**). Chol., cholesterol. Erg, ergosterol.
(PPT)

S5 Fig Alignment of *L*. *major* and *L*. *donovani* CYP51. **A**, Clustal Omega alignment. β 1–1 and 1–2 helices are positioned as in [12]. **B**, Secondary structure alignment. 3-D models of *L*. *major* and *L*. *donovani* CYP51 were generated using the I-TASSER server. The top scoring models were overlaid using UCSF Chimera. Red, *L*. *donovani*. Blue, *L*. *major*.
(PPT)

S1 Table Oligonucleotides used in this study. (XLS)

S2 Table Susceptibility of *L*. *major* to CYP51 inhibitors (EC50, μM). (DOC)

S3 Table Susceptibility of intracellular *L*. *donovani* amastigotes to select CYP51 inhibitors. (DOC)
