# Supplementary material for: Targeting Ergosterol Biosynthesis in Leishmania donovani: Essentiality of Sterol 14alpha-demethylase
Source: PLoS Negl Trop Dis. 2015 Mar 13;9(3):e0003588. doi: 10.1371/journal.pntd.0003588 (PMC4359151; doi:10.1371/journal.pntd.0003588)
Supplement: S1 Methods — (DOC) [file pntd.0003588.s001.doc]

**Supplemental methods**

**Chemical synthesis**

All reaction solvents were purified before use. Dichloromethane, tetrahydrofuran, and toluene were purified by passing through a column of activated A-1 alumina. All other reagents purchased from commercial suppliers were used as received. All reactions sensitive to moisture or oxygen were conducted under an argon atmosphere using flame-dried (under vacuum) or oven-dried (overnight) glassware. Removal of solvents was accomplished by using a rotary evaporator under reduced pressure in a water bath below 35°C, followed by exposure to high vacuum using a vacuum pump.

Proton nuclear magnetic resonance (^1^H NMR) spectra and carbon (^13^C) NMR spectra were recorded on a commercially available NMR spectrometer at 400 MHz and 176 MHz, respectively. The proton signal for non-deuterated solvent (δ 7.26 for CHCl_3_ or δ 2.50 for DMSO) was used as an internal reference for ^1^H NMR chemical shifts. Coupling constants (*J*) are reported in Hertz (Hz). ^13^C chemical shifts are reported relative to the δ 77.16 resonance of CDCl_3_ or the δ 39.52 resonance of DMSO-d_6_.

Analytical thin layer chromatography (TLC) was performed using glass plates precoated with a 0.25-mm thickness of silica gel. The TLC plates were visualized with UV light. Column chromatography was performed using a Biotage® Isolera flash purification system using Biotage® SNAP HP-SIL cartridge (30 μm silica, 10 g to 100 g size). Unless noted otherwise, all compounds isolated by flash chromatography were sufficiently pure by ^1^H NMR analysis for use in subsequent reactions. Polar compounds were purified using preparative high performance liquid chromatography (HPLC) using SunFire column (30 mm × 250 mm) with a linear gradient elution ranging from 10% to 100% of CH_3_CN/CH_3_OH (1/1) in H_2_O (containing 0.1% TFA) at 60 mL/min flow rate for 10 min followed by 100% of CH_3_CN/CH_3_OH (1/1) for additional 2 min.

The purity of all final compounds (typically ≥98%) was assayed at 254 nm wavelength by using analytical HPLC (Varian 1100 series) on a reverse phase ZORBAX Eclipse XDB-C18 column (4.6 × 150 mm, 5 μm). A linear gradient elution ranging from 2% to 98% CH_3_CN and H_2_O (containing 0.1% TFA and 1% CH_3_CN) at 1.5 mL/min was used. Compounds were lyophilized before dissolution in DMSO to give 10 mM stock solutions for use in biochemical and cell-based assays.

General procedure for the synthesis of inhibitors **1**, **2**, **3**, **4**, **5**, **6**, **7**, **8**, **9**, and **10**. To a solution of the appropriate benzoic acid (ca.1.2 eq), PyBOP (ca. 1.4 eq) and HOBt (ca. 10 mol%) in dry CH_2_Cl_2_ (5 mL) was slowly added triethylamine (ca. 4 eq.) at ambient temperature. The reaction mixture was stirred for 15 min until it became homogenous. L- or D-tryptophan derivative **21** was added, and the reaction mixture was stirred at room temperature for 1 h. After confirming that the reaction was complete by using TLC analysis, the solvent was removed under reduced pressure. Ethyl acetate (10 mL) was added to the crude product mixture, and this solution was washed then with saturated aqueous NaHCO_3_ (2 mL × 2) and brine (2 mL × 2). The organic layer was concentrated *in vacuo* and the crude product was directly subjected high performance liquid chromatographic purification to provide the titled products.

Compounds **1**, **2**, **6**, **8**, **9**, and **10** were reported previously. (.

*(R)-N-(3-(1H-Indol-3-yl)-1-oxo-1-(pyridin-4-ylamino)propan-2-yl)-3,5'-difluoro-2'-(trifluoromethyl)-[1,1'-biphenyl]-4-carboxamide TFA (****3****).* The general procedure was followed using carboxylic acid **13** as the acylating agent to afford **3** as a white solid (10%): ^1^H NMR (400 MHz, DMSO-d_6_) δ 11.56 (s, 1H), 10.92 (d, J = 2.5 Hz, 1H), 8.88 (dd, J = 6.9, 2.5 Hz, 1H), 8.75 – 8.60 (m, 2H), 8.12 – 7.99 (m, 2H), 7.96 (dd, J = 8.9, 5.4 Hz, 1H), 7.76 – 7.59 (m, 2H), 7.53 (td, J = 8.5, 2.7 Hz, 1H), 7.40 – 7.29 (m, 4H), 7.26 (dd, J = 8.1, 1.6 Hz, 1H), 7.06 (ddd, J = 8.1, 7.0, 1.2 Hz, 1H), 6.96 (ddd, J = 8.0, 6.9, 1.1 Hz, 1H), 5.02 – 4.81 (m, 1H), 3.44 – 3.16 (m, 2H); MS (ESI) *m/z* 565.2 [M+H]^+^.

*(R)-N-(3-(1H-Indol-3-yl)-1-oxo-1-(pyridin-4-ylamino)propan-2-yl)-3-fluoro-4'-methoxy-3'-(trifluoromethyl)-[1,1'-biphenyl]-4-carboxamide TFA (****4****).* The general procedure was followed using carboxylic acid **14** as the acylating agent to afford **4** as a white solid (32%): ^1^H NMR (400 MHz, DMSO-d_6_) δ 11.53 (s, 1H), 10.95 (d, J = 2.5 Hz, 1H), 8.69 (dd, J = 6.7, 3.6 Hz, 3H), 8.13 – 7.98 (m, 3H), 7.95 (d, J = 2.3 Hz, 1H), 7.79 – 7.61 (m, 4H), 7.39 (d, J = 8.9 Hz, 1H), 7.34 (d, J = 8.1 Hz, 1H), 7.29 (d, J = 2.4 Hz, 1H), 7.06 (ddd, J = 8.1, 6.9, 1.2 Hz, 1H), 7.01 – 6.87 (m, 1H), 4.90 (dt, J = 8.6, 6.1 Hz, 1H), 3.95 (s, 3H), 3.44 – 3.18 (m, 2H). ^13^C NMR (176 MHz, DMSO-d_6_) δ 172.81, 163.59, 160.69, 159.27, 157.38, 151.53, 143.85, 143.10, 143.06, 136.09, 132.72, 130.97, 130.95, 129.80, 127.08, 125.19, 125.15, 124.35, 124.13, 122.80, 122.23, 122.22, 121.22, 121.14, 121.05, 118.37, 118.33, 117.57, 117.40, 114.01, 113.87, 113.64, 111.42, 109.00, 56.46, 55.69, 26.99; MS (ESI) *m/z* 578.0 [M+H]^+^.

*(R)-N-(3-(1H-Indol-3-yl)-1-oxo-1-(pyridin-4-ylamino)propan-2-yl)-3-fluoro-[1,1':4',1''-terphenyl]-4-carboxamide TFA (****5****).* The general procedure was followed using carboxylic acid **15** as the acylating agent to afford **5** as a white solid (40%): ^1^H NMR (400 MHz, DMSO-d_6_) δ 11.54 (s, 1H), 10.95 (d, J = 2.5 Hz, 1H), 8.81 – 8.62 (m, 3H), 8.11 – 7.99 (m, 2H), 7.91 – 7.78 (m, 4H), 7.77 – 7.63 (m, 6H), 7.50 (dd, J = 8.4, 6.9 Hz, 2H), 7.44 – 7.37 (m, 1H), 7.34 (d, J = 8.1 Hz, 1H), 7.31 (d, J = 2.4 Hz, 1H), 7.06 (ddd, J = 8.2, 6.9, 1.2 Hz, 1H), 7.01 – 6.93 (m, 1H), 4.91 (dt, J = 8.5, 6.2 Hz, 1H), 3.42 – 3.21 (m, 2H); MS (ESI) *m/z* 555.5 [M+H]^+^.

*(R)-N-(3-(1H-indol-3-yl)-1-oxo-1-(pyridin-4-ylamino)propan-2-yl)-3,5'-difluoro-2'-methyl-[1,1'-biphenyl]-4-carboxamide TFA (****7****).* The general procedure was followed using carboxylic acid **17** as the acylating agent to afford **7** as a white solid (47%): ^1^H NMR (400 MHz, DMSO-d_6_) δ 11.62 (s, 1H), 10.94 (d, J = 2.5 Hz, 1H), 8.77 (dd, J = 6.8, 3.0 Hz, 1H), 8.73 – 8.62 (m, 2H), 8.11 – 7.98 (m, 2H), 7.66 (t, J = 7.7 Hz, 2H), 7.39 – 7.30 (m, 3H), 7.28 (dd, J = 7.9, 1.6 Hz, 1H), 7.17 (td, J = 8.6, 2.8 Hz, 1H), 7.13 – 7.02 (m, 2H), 6.96 (ddd, J = 8.0, 6.9, 1.1 Hz, 1H), 4.99 – 4.86 (m, 1H), 3.45 – 3.19 (m, 2H), 2.21 (s, 3H). ^13^C NMR (176 MHz, DMSO-d_6_) δ 172.82, 163.72, 161.04, 159.81, 159.66, 158.39, 158.11, 157.93, 151.66, 144.79, 144.74, 143.72, 140.85, 140.81, 136.09, 132.33, 132.28, 130.97, 130.96, 130.23, 130.22, 127.07, 125.14, 125.12, 124.15, 121.76, 121.68, 121.04, 118.42, 118.32, 116.81, 116.67, 115.97, 115.84, 114.83, 114.72, 114.43, 111.41, 109.02, 55.69, 26.98, 19.24; MS (ESI) *m/z* 511.2 [M+H]^+^.

General procedure synthesis of biphenyl-4-carboxylic acids **11, 12, 13**, **14**, **15**, **16**, and **17**. A reaction mixture of 4-bromo-2-fluorobenzoic acid (ca. 0.10 g, 0.46 mmol), arylboronic acid (1.1 eq), Pd_2_(dba)_3_ (3 mol%), PCy_3_ (6 mol %), and K_3_PO_4_ (2 M, 1 mL) in dioxane (4 mL) was stirred under microwave heating (100 °C) for 1 h. The palladium catalyst was removed by filtration through a pad of Celite, which was subsequently washed with ethyl acetate. The filtrate was acidified with 2N HCl (aq). The product mixture was diluted with ethyl acetate (30 mL) and washed with water (10 mL × 2) and brine (10 mL × 2). The organic layer was dried over magnesium sulfate, filtered, and concentrated in vacuo. The resulting crude product was purified by flash chromatography to afford the 3-fluoro-biphenyl-4-carboxylic acids.

Compounds **11**, 1**2**, 1**6, 18a**, **18b**, **19a**, **19b**, **20a**, and **20b** were reported previously

*3,5'-Difluoro-2'-(trifluoromethyl)-[1,1'-biphenyl]-4-carboxylic acid (****13****).* The general procedure was followed using (5-fluoro-2-(trifluoromethyl)phenyl)boronic acid to provide **13** as a white solid (92%): ^1^H NMR (400 MHz, DMSO-d_6_) δ 13.42 (s, 1H), 8.02 – 7.84 (m, 2H), 7.52 (td, J = 8.5, 2.7 Hz, 1H), 7.42 (dd, J = 9.2, 2.7 Hz, 1H), 7.35 (dd, J = 11.4, 1.6 Hz, 1H), 7.28 (dd, J = 8.1, 1.5 Hz, 1H). ^13^C NMR (101 MHz, DMSO-d_6_) δ 164.74, 164.71, 164.67, 162.17, 161.56, 159.00, 144.04, 143.95, 141.32, 141.23, 131.56, 129.38, 129.33, 129.28, 127.73, 125.00, 123.51, 123.48, 123.21, 123.18, 122.29, 119.23, 119.15, 119.12, 118.92, 117.61, 117.37, 115.96, 115.74; MS (ESI) *m/z* 303.1 [M+H]^+^.

*3-Fluoro-4'-methoxy-3'-(trifluoromethyl)-[1,1'-biphenyl]-4-carboxylic acid (****14****).* The general procedure was followed using (4-methoxy-3-(trifluoromethyl)phenyl)boronic acid to provide **14** as a white solid (88%): ^1^H NMR (400 MHz, DMSO-d_6_) δ 13.25 (s, 1H), 8.07 (dd, J = 8.8, 2.4 Hz, 1H), 7.96 (d, J = 2.4 Hz, 1H), 7.92 (t, J = 8.0 Hz, 1H), 7.71 (dd, J = 12.4, 1.8 Hz, 1H), 7.65 (dd, J = 8.1, 1.8 Hz, 1H), 7.39 (d, J = 8.8 Hz, 1H), 3.95 (s, 3H). ^13^C NMR (101 MHz, DMSO-d_6_) δ 164.81, 164.78, 162.92, 160.37, 157.50, 144.62, 144.53, 132.79, 132.54, 129.67, 125.36, 125.31, 125.25, 125.20, 124.90, 122.19, 122.15, 122.12, 117.83, 117.72, 117.65, 117.34, 117.04, 114.72, 114.49, 113.62, 56.43; MS (ESI) *m/z* 315.1 [M+H]^+^.

*3-Fluoro-[1,1':4',1''-terphenyl]-4-carboxylic acid (****15****).* The general procedure was followed using [1,1'-biphenyl]-4-ylboronic acid to provide **15** as a white solid (84%): ^1^H NMR (400 MHz, DMSO-d_6_) δ 13.26 (s, 1H), 7.96 (t, J = 8.2 Hz, 1H), 7.92 – 7.86 (m, 2H), 7.84 – 7.78 (m, 2H), 7.78 – 7.66 (m, 4H), 7.54 – 7.46 (m, 2H), 7.44 – 7.36 (m, 1H). ^13^C NMR (101 MHz, DMSO-d_6_) δ 164.87, 164.84, 162.98, 160.42, 145.88, 145.80, 140.54, 139.26, 136.53, 132.61, 129.06, 127.85, 127.60, 127.34, 126.70, 122.33, 122.30, 117.87, 117.77, 114.80, 114.57; MS (ESI) *m/z* 293.1 [M+H]^+^.

*3,5'-Difluoro-2'-methyl-[1,1'-biphenyl]-4-carboxylic acid (****17****).* The general procedure was followed using (5-fluoro-2-methylphenyl)boronic acid to provide **17** as a white solid (96%): ^1^H NMR (400 MHz, DMSO-d_6_) δ 13.25 (s, 1H), 7.92 (t, J = 7.9 Hz, 1H), 7.41 – 7.32 (m, 2H), 7.30 (dd, J = 8.0, 1.6 Hz, 1H), 7.22 – 7.07 (m, 2H), 2.21 (s, 3H). ^13^C NMR (101 MHz, DMSO-d_6_) δ 164.88, 164.84, 162.13, 161.58, 159.57, 159.17, 146.52, 146.43, 140.75, 140.67, 132.37, 132.29, 131.88, 131.00, 130.97, 125.17, 125.14, 118.21, 118.11, 117.60, 117.38, 116.03, 115.82, 115.00, 114.79, 19.25; MS (ESI) *m/z* 249.1 [M+H]^+^.

***Leishmania* High Content Assay**

Screening was performed as described in . Briefly, 5x10^4^ THP-1 macrophages were seeded in RPMI media in the presence of 0.1 µM phorbol myristate acetate (PMA, Sigma) at 37°C for 48 h in 96-well plates. Cells were then washed and stationary phase *L. donovani* promastigotes added at a 15:1 parasite to macrophage ratio (7.5x10^5^ parasites/well) for 5 h. After 4 h incubation at 37°C, non-internalized promastigotes were removed by three successive washes with RPMI, followed by one wash with RPMI supplemented with 5% horse serum. Compounds were added at 10 μM final concentration and resulting plates were incubated at 37°C for 72 h. Cells were then fixed with 4% formaldehyde and stained with 4′,6′-diamidino-2-phenylindole (DAPI). Images were obtained with an automated InCell 2000 automated imaging system (G.E. Healthcare). A short exposure was taken to acquire nuclei in the linear range of the CCD and a long exposure to acquire *Leishmania*  parasites which stain less well. The host cell nuclei and *Leishmania* were masked with object segmentation. A boundary mask for each nuclei and its nearby parasite were created by repeating the *Leishmania* segmentation at a higher sensitivity, which also masks small features of the nuclei. The numerous small masks were merged and separated into cell boundaries by dilating, clump breaking with host cell as the seed, and eroding. The numbers of parasite inside the boundary, but outside the nucleus, were counted and the counts of parasite per cell used to bin cells into different levels of infection. Normalized activity was calculated as 1-(sample infection ratio- μ_AmpB_)/( μ_DMSO_−μ_AmpB_), with μ_DMSO_ the average infection ratio of the 1% DMSO negative control and μ_AmpB_  the average infection ratio of the amphotericin B positive control. Infection ratio was calculated as follows: number of infected THP-1/total number of THP-1 cells.

***L. major* CYP51 inhibitor assay**

CYP51 inhibitors were tested in dose response on *L. major* as described above with the following modifications: *L. major* metabolized resazurin slower than *L. donovani.* Parasites were therefore incubated in the presence of resazurin for 72 h rather than 5 h. This increased exposure time to compounds from 3 days to 6 days.

**CYP51 secondary structure modeling and alignment**

CYP51 crystal structures are not available for *L. major* and *L. donovani.* CYP51 structure modeling was performed using the I-TASSER server version 4.2 with default parameters , using CYP51 sequences for *L. major* (LmjF.11.1100) and *L. donovani* (LdBPK_111100.1) retrieved from TriTrypDB . Structural alignments of the top-scoring models for both parasites was performed using UCSF chimera .

**Supplemental references**

1. Calvet CM, Vieira DF, Choi JY, Kellar D, Cameron MD, et al. (2014) 4-Aminopyridyl-based CYP51 inhibitors as anti-Trypanosoma cruzi drug leads with improved pharmacokinetic profile and in vivo potency. J Med Chem 57: 6989-7005.

2. Choi JY, Calvet CM, Gunatilleke SS, Ruiz C, Cameron MD, et al. (2013) Rational development of 4-aminopyridyl-based inhibitors targeting Trypanosoma cruzi CYP51 as anti-chagas agents. J Med Chem 56: 7651-7668.

3. Choi JY, Calvet CM, Vieira DF, Gunatilleke SS, Cameron MD, et al. (2014) R-Configuration of 4-Aminopyridyl-Based Inhibitors of CYP51 Confers Superior Efficacy Against Trypanosoma cruzi. Acs Med Chem Letters 5: 434-439.

4. De Muylder G, Ang KKH, Chen S, Arkin MR, Engel JC, et al. (2011) A Screen against Leishmania Intracellular Amastigotes: Comparison to a Promastigote Screen and Identification of a Host Cell-Specific Hit. Plos Negl Trop Dis 5.

5. Zhang Y (2008) I-TASSER server for protein 3D structure prediction. Bmc Bioinformatics 9: 40.

6. Aslett M, Aurrecoechea C, Berriman M, Brestelli J, Brunk BP, et al. (2010) TriTrypDB: a functional genomic resource for the Trypanosomatidae. Nucleic Acids Res 38: D457-D462.

7. Pettersen EF, Goddard TD, Huang CC, Couch GS, Greenblatt DM, et al. (2004) UCSF Chimera--a visualization system for exploratory research and analysis. J Comput Chem 25: 1605-1612.
