# Supplementary figures and images for: Targeting Ergosterol Biosynthesis in Leishmania donovani: Essentiality of Sterol 14alpha-demethylase
Source: PLoS Negl Trop Dis. 2015 Mar 13;9(3):e0003588. doi: 10.1371/journal.pntd.0003588 (PMC4359151; doi:10.1371/journal.pntd.0003588)

## Slide 1
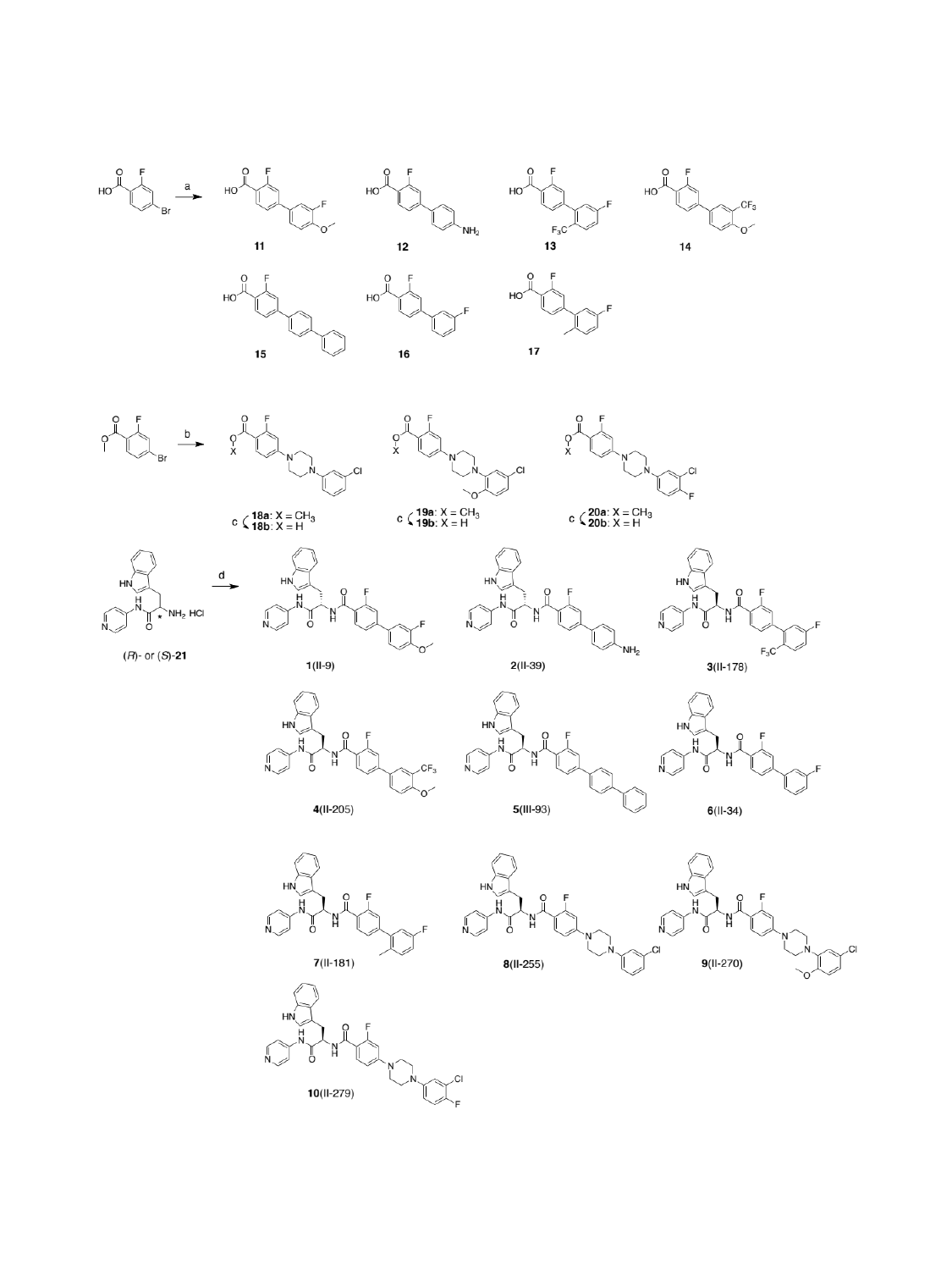

#

Supplement: S1 Scheme — Reagents and conditions: (a) Arylboronic acid, 5 mol% Pd2(dba)3, 10 mol% PCy3, 2M K3PO4, dioxane, 100°C (microwave), 1h, ca. 90% (b) 1-(aryl)piperazine, Pd(OAc)2, P(o-tolyl)3, Cs2CO3, toluene, 50°C, 48 h, ca. 70% (c) 10% NaOH (aq), MeOH/THF (1/1), 60°C, 3 h, ca. 95% (d) 11, 12, 13, 14, 15, 16, 17, 18b, 19b, and 20b (as appropriate), PyBOP, HOBt, Et3N, CH2Cl2, 23°C, 1h, ca. 50%. (PPT) [file pntd.0003588.s002.ppt]

## Slide 1
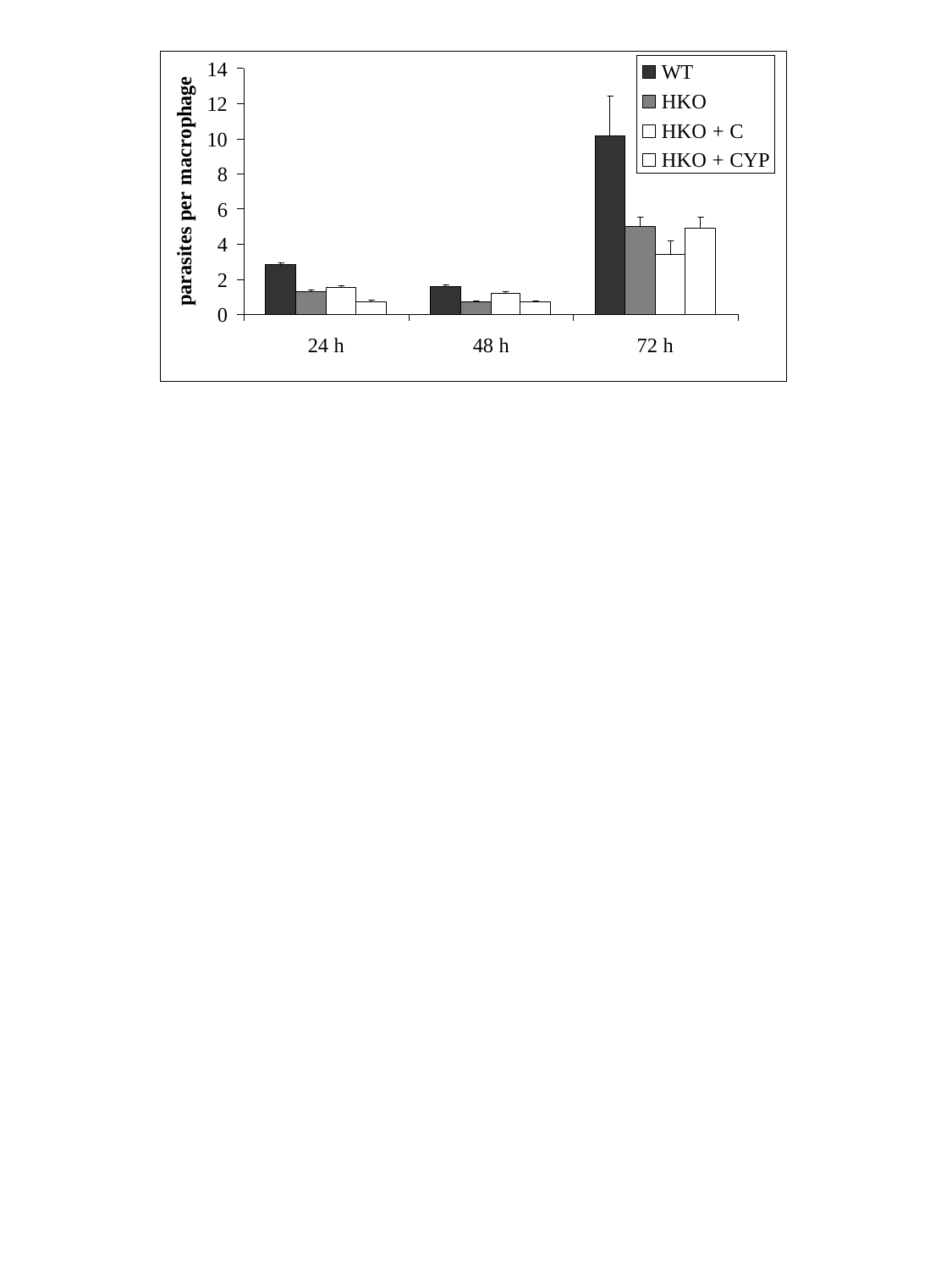

#

Supplement: S1 Fig — THP1 macrophages were infected at a 10:1 parasite to macrophage ratio. Cells were fixed and stained with DAPI 24, 48 and 72 h post-infection, and parasite numbers per infected cell were determined by automated high-throughput imaging and parasite detection (PPT) [file pntd.0003588.s003.ppt]

## Slide 1
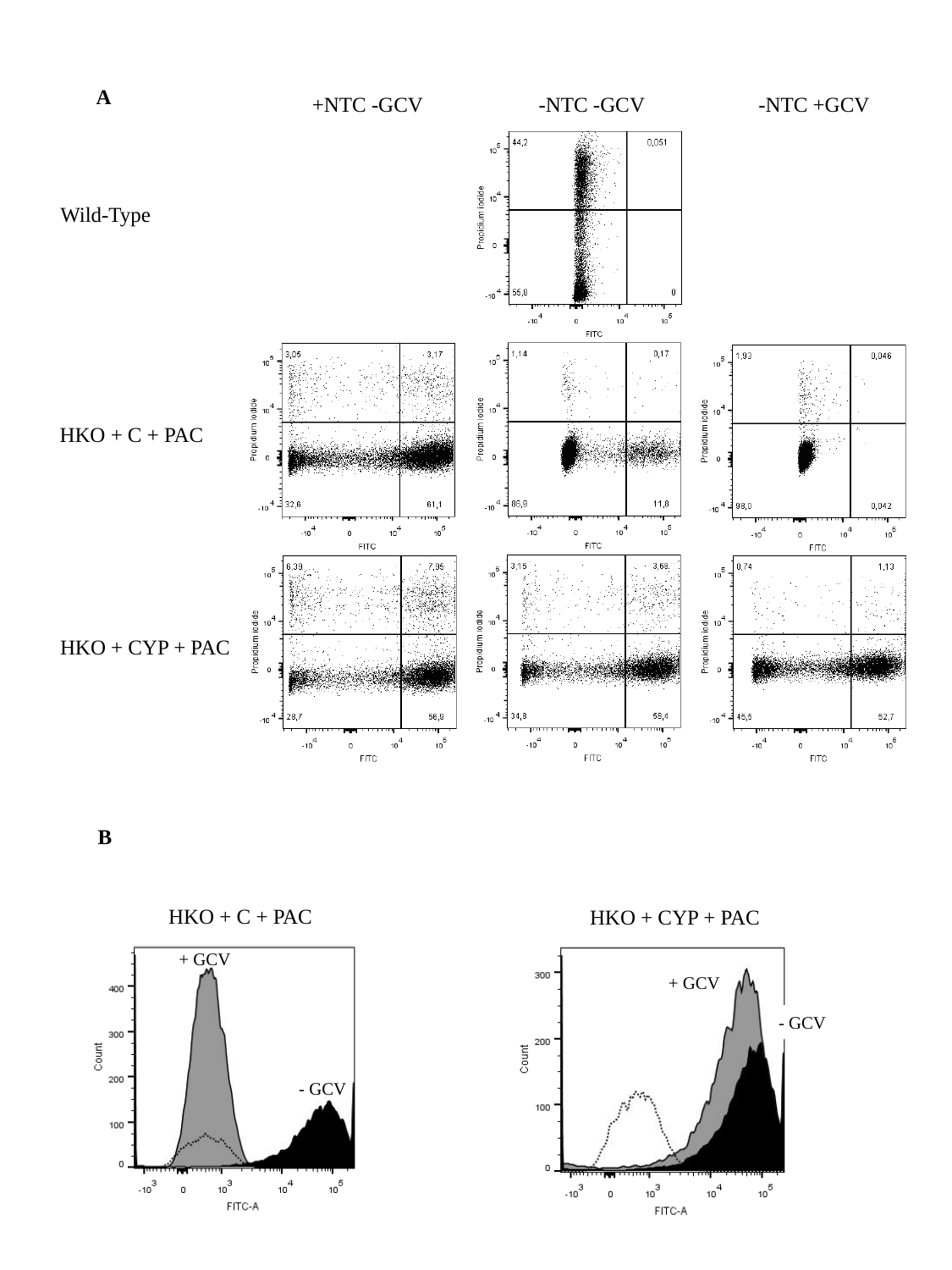

A
+NTC -GCV
-NTC -GCV
-NTC +GCV
Wild-Type
HKO + C + PAC
HKO + CYP + PAC
B
HKO + C + PAC
HKO + CYP + PAC
+ GCV
+ GCV
- GCV
- GCV

Supplement: S3 Fig — Parasites were treated with NTC (positive selection), GCV (negative selection) or left untreated (-NTC-GCV) for five weeks. One representative cell line is shown for HKO + C + PAC and for HKO + CYP + PAC. A, Quadrant analysis. Numbers indicate the percentage of cells in each quadrant. B, Representative GFP histogram plots of PI-negative cells. Wild-type parasites (dotted line) serve as the non-fluorescent cutoff reference. Black, NTC treatment (positive selection). Grey, GCV treatment (negative selection). (PPT) [file pntd.0003588.s005.ppt]

## Slide 1
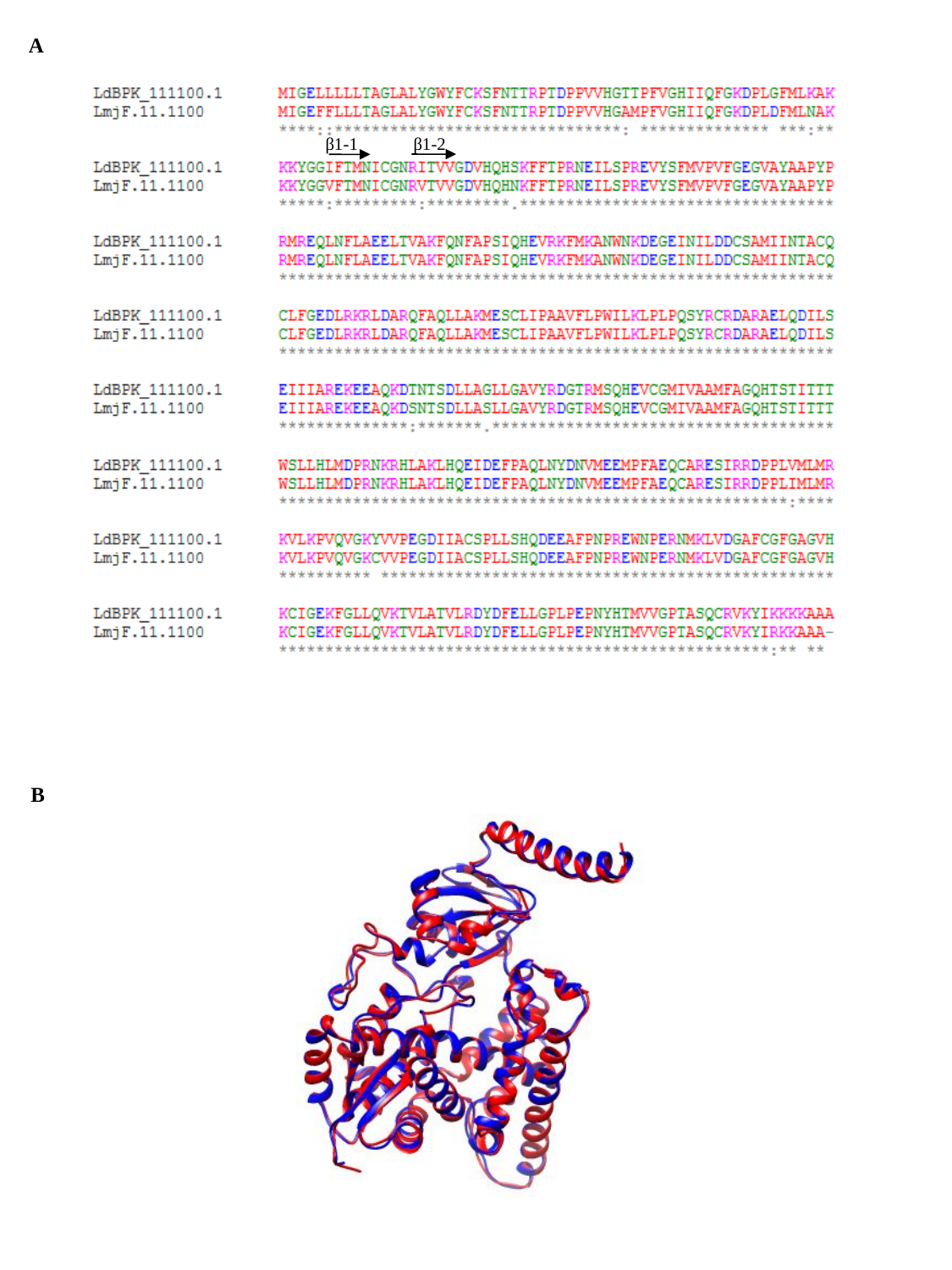

A
#
β1-1
β1-2
B

Supplement: S5 Fig — A, Clustal Omega alignment. β 1–1 and 1–2 helices are positioned as in [12]. B, Secondary structure alignment. 3-D models of L. major and L. donovani CYP51 were generated using the I-TASSER server. The top scoring models were overlaid using UCSF Chimera. Red, L. donovani. Blue, L. major. (PPT) [file pntd.0003588.s007.ppt]
