# Supplementary material for: Targeting Ergosterol Biosynthesis in Leishmania donovani: Essentiality of Sterol 14alpha-demethylase
Source: PLoS Negl Trop Dis. 2015 Mar 13;9(3):e0003588. doi: 10.1371/journal.pntd.0003588 (PMC4359151; doi:10.1371/journal.pntd.0003588)
Supplement: S2 Fig — Correct targeting of the hygromycin (A) and puromycin (B) resistance knockout cassettes was verified by PCR using one primer upstream of CYP51 and one specific to the resistance marker (primers 7 and 8 (hygromycin) or 7 and 9 (puromycin)). HKO1 + CYP + PAC clones 2, 4, 5; HKO2 + C + PAC clones 1, 2 and 3; HKO2 + CYP + PAC clones 1, 2, 4 and 5 have correct targeting of both knockout cassettes. (PPT) [file pntd.0003588.s004.ppt]

## Slide 1
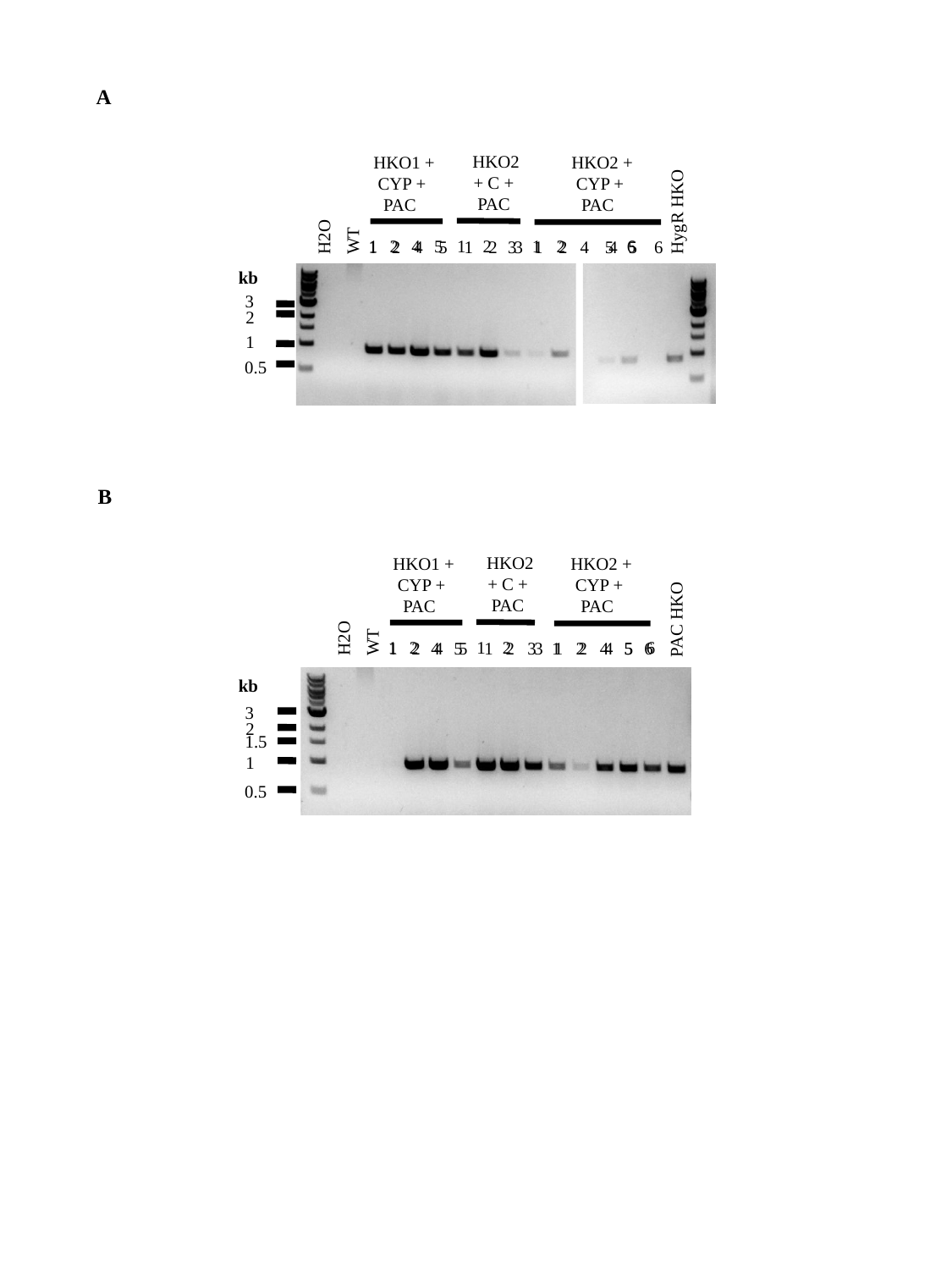

HKO2 + C + PAC
 HKO1 + CYP + PAC
 HKO2 + CYP + PAC
WT
H2O
HygR HKO
2
1
1
2
4
5
6
1
2
4
5
3
1
2
4
5
1
2
3
1
2
4
5
6
kb
3
2
1
0.5
A
H2O
PAC HKO
2
1
1
2
4
5
6
1
2
4
5
3
 HKO2 + C + PAC
 HKO1 + CYP + PAC
 HKO2 + CYP + PAC
WT
kb
3
2
1.5
1
0.5
1
2
4
5
1
2
3
1
2
4
5
6
B
