# Supplementary material for: Targeting Ergosterol Biosynthesis in Leishmania donovani: Essentiality of Sterol 14alpha-demethylase
Source: PLoS Negl Trop Dis. 2015 Mar 13;9(3):e0003588. doi: 10.1371/journal.pntd.0003588 (PMC4359151; doi:10.1371/journal.pntd.0003588)
Supplement: S4 Fig — CYP51 persistence was assessed by qPCR (A) and Western blot (B) following seven weeks of GCV selection. Sterol profiles and ergosterol levels were determined by GC-MS (C). Chol., cholesterol. Erg, ergosterol. (PPT) [file pntd.0003588.s006.ppt]

## Slide 1
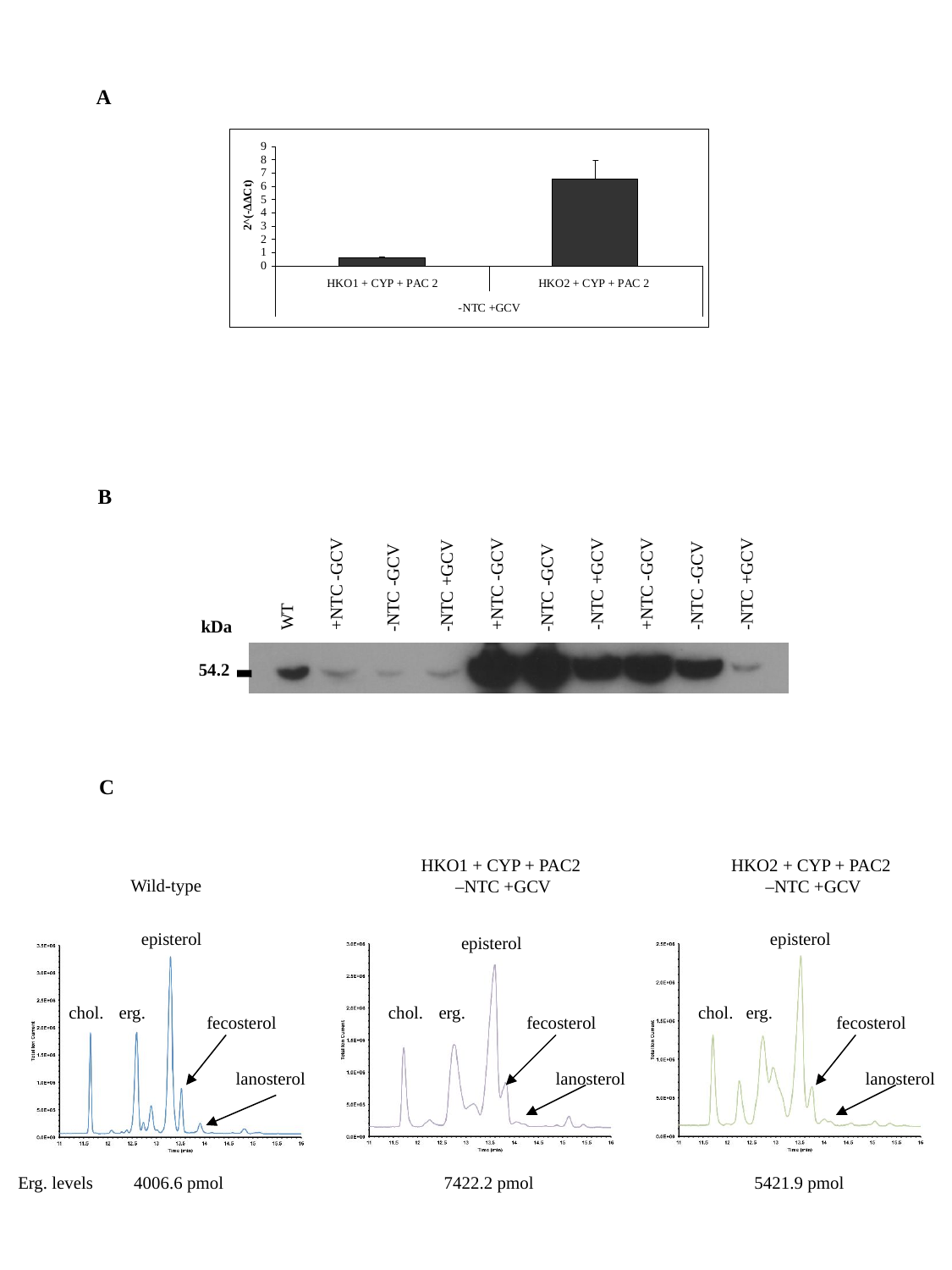

#
A
B
+NTC -GCV
+NTC -GCV
-NTC +GCV
+NTC -GCV
-NTC +GCV
-NTC +GCV
-NTC -GCV
-NTC -GCV
-NTC -GCV
WT
kDa
54.2
C
HKO2 + CYP + PAC2
 –NTC +GCV
HKO1 + CYP + PAC2
 –NTC +GCV
Wild-type
episterol
episterol
episterol
chol.
erg.
chol.
erg.
chol.
erg.
fecosterol
fecosterol
fecosterol
lanosterol
lanosterol
lanosterol
Erg. levels
4006.6 pmol
7422.2 pmol
5421.9 pmol
