# Supplementary material for: Targeting Ergosterol Biosynthesis in Leishmania donovani: Essentiality of Sterol 14alpha-demethylase
Source: PLoS Negl Trop Dis. 2015 Mar 13;9(3):e0003588. doi: 10.1371/journal.pntd.0003588 (PMC4359151; doi:10.1371/journal.pntd.0003588)
Supplement: S2 Table — (DOC) [file pntd.0003588.s009.doc]

**Table S2. Susceptibility of *L. major* to CYP51 inhibitors (EC50, μM)**

| **Structure** | **Name** | ***T. cruzi***  **cell-based (μM)** | **WT *L. donovani*** ^a^ | **WT *L. major*** ^b^ |
| --- | --- | --- | --- | --- |
| **No significant difference** |  |  |  |  |
|  | 1 (II-9) | 0.47±0.6 | >10.0 | >10.0 |
| 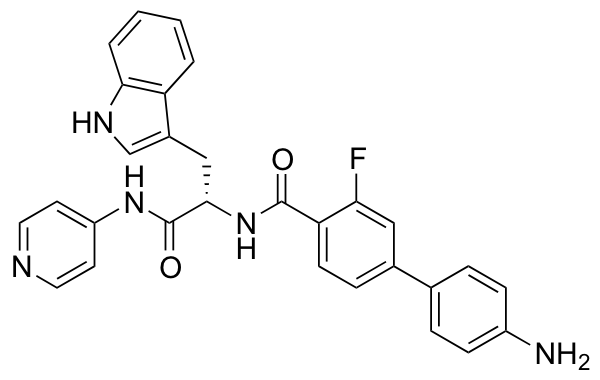 | 2 (II-39) | 0.23±0.13 | >10.0 | >10.0 |
|  | 3 (II-178) | 0.00044±0.00016 | >10.0 | >10.0 |
|  | 4 (CYP-II-205) | 1.2±0.5 | 9.25±0.20 | >10.0 |
|  | 5 (III-93) | 0.027±0.027 | 6.92±0.31 | >10.0 |

| **Decreased activity on HKO+CYP** |  |  |  |  |
| --- | --- | --- | --- | --- |
| 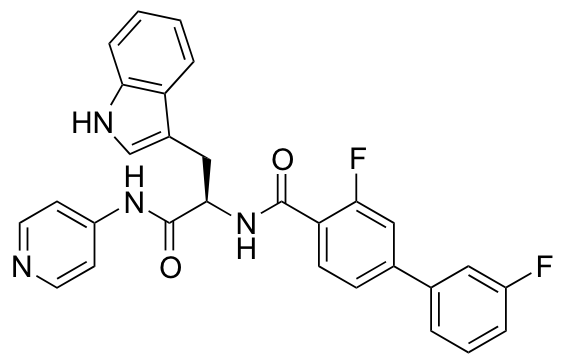 | 6 (II-34) | 0.00012±0.00039 | >10.0 | >10.0 |
|  | 7 (II-181) | 0.0033±0.0001 | 8.64±0.61 | >10.0 |
|  | 8 (II-255) | 0.0068±0.0019 | 8.69±0.20 | >10.0 |
|  | 9 (II-270) | 0.017±0.009 | 6.25±0.31 | >10.0 |
|  | 10 (II-279) | 0.00064±0.0008 | 8.59±0.25 | >10.0 |

^a^ 3 day exposure to compounds

^b^ 6 day exposure to compounds
