# Supplementary material for: Targeting Ergosterol Biosynthesis in Leishmania donovani: Essentiality of Sterol 14alpha-demethylase
Source: PLoS Negl Trop Dis. 2015 Mar 13;9(3):e0003588. doi: 10.1371/journal.pntd.0003588 (PMC4359151; doi:10.1371/journal.pntd.0003588)
Supplement: S3 Table — (DOC) [file pntd.0003588.s010.doc]

**Table S3. Susceptibility of intracellular *L. donovani* amastigotes to select CYP51 inhibitors**

| **Structure** | **Name** | **% inhibition in preliminary screen (96WP, 10 μM)** | **% inhibition in secondary screen (384 WP, 20 μM)** | |
| --- | --- | --- | --- | --- |
|  | 1 (II-9) | 80.7 | 4 ± 7 | |
| 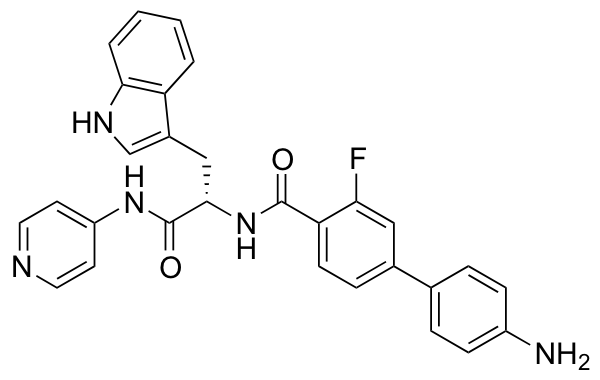 | 2 (II-39) | 81.2 | 6 ±16 | |
|  | 3 (II-178) | 72.0 | 25 ± 8 | |
|  | 4 (CYP-II-205) | 86.8 | 41 ± 6 | |
|  | 5 (III-93) | 84.4 | 49 ± 1 | |
| 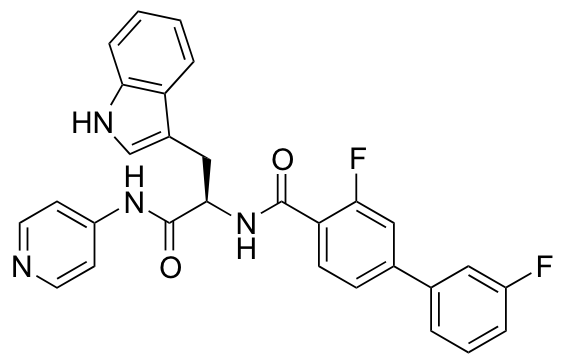 | 6 (II-34) | 64.3 | 17 ± 16 |  |
|  | 7 (II-181) | 90.4 | 29 ± 13 |  |
|  | 8 (II-255) | 72.7 | 30 ± 9 |  |
|  | 9 (II-270) | 61.6 | 18 ± 3 |  |
|  | 10 (II-279) | 75.3 | 15 ± 15 |  |
